# Public expenditure on road safety policy and programmes: right or wrong direction to achieve the decade of action on road safety goal in Thailand

**DOI:** 10.1186/1471-2458-14-S1-O18

**Published:** 2014-01-29

**Authors:** Pudtan Phanthunane, Jirakom Sirisrisakulchai, Thaweesak Taekratoke, Supasit Pannarunothai

**Affiliations:** 1 Centre for Health Equity Monitoring, Naresuan University, Phitsanulok 65000, Thailand

## Background

The objectives of the study were (1) to explore the pattern of public spending on road safety programmes corresponding to 5-E strategy (i.e. evaluation, engineering, education, enforcement and emergency medical service), and (2) to investigate road safety policy directions.

## Materials and methods

The government budget documents from 5 ministries including Ministry of Transport, Education, Interior, Public Health and Royal Thai Police were reviewed. Two researchers identified budget used in road safety projects with 5-E strategies independently; the kappa analysis was used to test inter-rater reliability. Information from Thai Health Promotion Foundation and Road Safety Fund was also gathered using a developed excel-based template. Semi-structured interviews were conducted among road safety experts. Some mathematical and statistical analyses were applied to evaluate the efficiency of road safety policy.

## Results

Thailand devoted around 18 billion Baht each year to prevent road traffic accidents. The majority of public budget was allocated to engineering strategies (50%), followed by enforcement (23%), emergency medical services (12%), evaluation (8%) and education (7%). The road safety experts, however, suggested that the strategies of education, enforcement and particularly evaluation are Thailand‘s key success factors. Thus, an area-based road safety information system should be developed. Data Envelopment Analysis suggested an inefficiency of law-enforcement system in Thailand by comparison with other countries worldwide.

## Conclusions

Despite insufficient data and evidence to clarify the outcomes of road safety policy and programmes, it is possible to conclude that (1) there is an underinvestment in road safety in Thailand and (2) the government allocates budget inappropriately. This study suggests allocating more public spending on road safety management data systems that empower local service providers to access and analyse current accident information. Investing in strategies of education and enforcement effectively influencing road user behaviour would be the right way to achieve the ultimate goal.

